# More on the MORE Life Experience Model: What We Have Learned (So Far)

**DOI:** 10.1007/s10790-018-9661-x

**Published:** 2018-09-28

**Authors:** Judith Glück, Susan Bluck, Nic M. Weststrate

**Affiliations:** 1 Department of Psychology, University of Klagenfurt, 9020 Klagenfurt, Austria; 2 University of Florida, Gainesville, FL USA; 3 University of Klagenfurt, Klagenfurt, Austria

We have all had difficult times and challenges in our lives, and most of us feel that we learned something from those experiences. At the same time, few people actually become wise in the course of their lives – while most of us become (or remain) well-adapted and happy, generally satisfied, or even bitter or depressed. Why is it that some people, but not others, grow wise over time by learning from life’s challenges (Linley & Joseph, 2004)? In the MORE Life Experience Model (Glück & Bluck, 2013), we argued that life challenges are catalysts for the development of wisdom, and that psychological resources crucially influence how people appraise life challenges, how they deal with them, and how they integrate them into their life story as time goes on. Based on the literature on wisdom and growth from challenging experiences, we proposed five resources as important for the development of wisdom: Mastery, Openness, Reflectivity, and Emotion Regulation including Empathy – in short, MORE.

Since proposing the model, we have conducted a first empirical test of its predictions. This paper describes our expected and unexpected findings, which provide insights that we integrate to further refine and elaborate the MORE Life Experience Model. First, we describe the theoretical and empirical background of the original model.

## The MORE Life Experience Model

### Life Challenges as Catalysts of Wisdom

Our first proposition was that life experiences that challenge a person’s beliefs and worldviews are: (a) the main life context in which wisdom manifests, and (b) necessary for the continual development of wisdom. When people are asked about situations in their life in which they did something wise, most describe an important, difficult situation such as a long-term life decision, a complicated social conflict, or a serious illness (Glück, Bluck, Baron, & McAdams, 2005). That is, wisdom is both manifest, and has a chance to further grow, when individuals face difficult obstacles or decisions that force them to question their own priorities and worldviews. We proposed specifically that use of the MORE resources should help individuals to manifest wisdom in the face of these challenging situations.

We also suggested that use of the MORE resources in reflecting on life challenges enables people to grow wiser. Life experience is agreed upon by laypeople and wisdom researchers (e.g., Ardelt, 2005; Baltes & Staudinger, 2000; Bluck & Glück, 2005; Glück & Bluck, 2011; Jeste, Ardelt, Blazer, Kraemer, Vaillant, & Meeks, 2010; Sternberg, 1998; Webster, 2003) as playing an important role in the continued development of wisdom. The MORE Life Experience Model draws specifically upon work suggesting that crises and obstacles can challenge people’s worldviews and thereby broaden their perspective (see also Ardelt; 2005; Kinnier, Tribbensee, Rose, & Vaughan, 2001; Kramer, 2003) and that particular psychological resources are essential for growth in the wake of adversity (e.g., Aldwin, 2007; Calhoun & Tedeschi, 2006; Joseph & Linley, 2005).

### The MORE Resources

We identified five resources that were repeatedly mentioned in the literature on life-span development, the development of wisdom, and growth from adversity, and are conceptually linked to growth in wisdom from difficult life experiences.

*Sense of mastery*. Most people have a healthy sense of illusory control that helps maintain stability and well-being (e.g., Peterson & Bossio, 2001; Taylor & Brown, 1988). Wise individuals, however, are more realistically aware of the uncertainty and unpredictability of life (Baltes & Staudinger, 2000; McKee & Barber, 1999) while also feeling that, having learned from experience, they will somehow be able to master whatever happens. Thus, mastery is a dialectical concept that combines full awareness of life’s uncontrollability and unpredictability with trust in one’s own ability to cope. Wise individuals are able to take action on things that they can control and accept things that they cannot control (Ardelt, 2005).

*Openness*. Wise individuals are interested in viewing situations from multiple perspectives (e.g., Staudinger, Lopez, & Baltes, 1997; Webster, 2003). They are non-judgmental, accept goals and values that differ from their own, and enjoy learning from others. They seek out new experiences and adapt well to the changes life inevitably brings. Webster (2003, 2007) considers openness as one of five components of wisdom itself. We propose, however, that openness is a precursor to wisdom (cf. Ardelt, 2011) because it enables people to learn from experiences and from others. Openness is certainly also a component of wisdom, and it is an interesting question in itself how wise individuals are able to maintain their openness to new ideas and experiences way into old age, where openness usually declines (Glück, in press). However, we believe that it is there before wisdom is – it paves the way for new experiences and new perspectives that become part of an individual’s wisdom-related knowledge and expertise.

*Reflectivity*. We define reflectivity as a person’s motivation to think about complex issues in a complex way. Reflective people look back on life experiences and think deeply about them. They are willing to question their own past and current views and behavior, as their goal is to develop a deeper understanding and not to reassure their own views. Reflection is a key ingredient of wisdom in laypeople’s conceptions (Bluck & Glück, 2005) and a component of Ardelt’s (2003, 2004) and Webster’s (2003, 2007) definitions of wisdom. We assume that being reflective, like being open, sets the stage for the development and growth of wisdom (Staudinger, 2001).

*Emotion regulation.* Wise individuals are attentive to their emotions, tolerant of ambivalent feelings, and able to manage emotion as fits the situation. Laypeople’s theories of wisdom often include calm in the face of conflict, which is arguably the most visible sign of emotion regulation (Bluck & Glück, 2005), and of particular importance when dealing with negative events (Troy, Wilhelm, Shallcross, & Mauss, 2010). As their aim is to understand life more fully, wise individuals neither suppress negative feelings nor dwell on them extensively (Kunzmann, 2004). They are also able to appreciate the positive things in life (König & Glück, 2014). As with openness, Webster’s model of wisdom (Webster, 2003, 2007) includes emotion regulation as a component, while we and Ardelt (2011) view it as a developmental precursor.

*Empathy.* We view empathy as an important precondition for the development of wisdom: those able to take others’ perspectives are more likely to develop a view of life that takes the needs of others and the common good into account (Jeste et al., 2010; Sternberg, 1998). Ardelt (2003) proposed compassion as the core of the affective dimension of wisdom. Such concern for others is also a component of wisdom in lay theories (Bluck & Glück, 2005): For wise individuals, concern for others is not limited to family or friends but includes a larger view of those in need of support, across humanity (Levenson, Jennings, Aldwin, & Shiraishi, 2005). Individuals popularly cited as wise have often created significant positive change in the world (Weststrate, Ferrari, & Ardelt, 2016).

## Testing the MORE Life Experience Model: the First Empirical Study

The MORE Life Experience Model argues that people higher in a sense of mastery, openness, reflectivity, emotion regulation, and empathy are more likely than others both to display wisdom in dealing with life challenges and to continue growing towards greater wisdom across a lifetime. A strict test of these developmental dynamics would require longitudinal data of individuals before, during and after naturally-occurring stressful life events. Given the difficulty and duration of that type of research, as a first step, we empirically tested the hypothesis that the MORE resources are statistically correlated with measures of wisdom in a cross-sectional design. In the following, we describe this study and its main findings.

### Methods

*Participants.* The fact that wisdom is a rare phenomenon makes it somewhat hard to investigate – as we have learned, recruiting a general-population sample yields few highly wise individuals. Therefore, we tried to increase the proportion of wise individuals in our sample by using a wisdom nomination approach. Through newspapers and radio programs, community members were invited to nominate any person that they felt was particularly wise. Excluding self-nominations, 82 people were nominated, and 47 of them agreed to participate. For comparison purposes, 123 other participants were recruited through invitation letters sent to a large population sample in the same region. Thus, the final sample consisted of 170 participants (90 women, 80 men), most of whom (86.5%) were 40 – 92 years of age. All participants came to the lab for two interview sessions and filled out some questionnaires at home (details are described in Glück et al., 2013). They were paid € 70 (about U.S. $80.00) for participation.

*Measures.* How wisdom is measured can affect results significantly. Most existing measures of wisdom are either self-report scales or open-ended responses to life problems. The two methods are not highly correlated (Glück, 2018; Glück et al., 2013). Self-report measures present a paradox: people who describe themselves as wise may lack the self-reflectivity that defines wisdom (Aldwin, 2009; Glück et al., 2013). Open-ended measures are not subject to such biases, but highly effort-consuming to analyze, and may overemphasize intellectual aspects of wisdom (Ardelt, 2004). We thus measured wisdom and the MORE resources using self-report measures in a relatively large sample and open-ended measures in a subsample, to ensure our results would not be an artifact of the methods used. The subsample consisted of the 47 wisdom nominees and 47 control participants parallel to the nominees in age and gender (for details, see Glück et al., 2013).

Participants completed three *self-report measures of wisdom*. The Self-Assessed Wisdom Scale (SAWS; Webster, 2007) measures five components of wisdom: openness, emotional regulation, humor, critical life experience, and reminiscence and reflectiveness. The Three-Dimensional Wisdom Scale (3D-WS; Ardelt, 2003) assesses cognitive, reflective, and compassionate dimensions of wisdom. The revised Adult Self-Transcendence Inventory (ASTI; Levenson et al., 2005) defines wisdom as self-transcendence. Reliabilities were satisfactory to excellent for all self-report measures (see Glück et al., 2013).

The *open-ended wisdom measure* came from the Berlin Wisdom Paradigm (BWP; Staudinger, Smith, & Baltes, 1994). After some practice with thinking aloud, participants were presented with a standard life-review problem: “In reflecting over their life, people sometimes realize that they have not achieved what they had once wanted to achieve. What could a person consider and do in such a situation?” Response transcripts were evaluated by trained raters following the BWP manual (Staudinger et al., 1994). There were two independent raters for each criterion: factual knowledge, procedural knowledge, life-span contextualism, value relativism, and recognition/management of uncertainty.

As *self-report measures of the MORE resources*, we used scales or relevant parts of scales from well-established measures. *Sense of Mastery* includes eight items from Wagnild and Young’s (1993) Resilience Scale referring to acceptance of uncontrollability (e.g., “I do not dwell on things that I can’t do anything about”) and dealing with hardship (“I can get through difficult times because I’ve experienced difficulty before”); Cronbach’s α was .76. *Openness* was assessed with the Openness scale of the NEO FFI (12 items, α = .78). For Reflectivity, five items were selected from the Psychological Mindedness Scale (Conte, Plutchik, Jung, Picard, Karasu, & Lotterman, 1990; e.g., “I often find myself thinking about what made me act in a certain way”) and the directive-function scale of the Talking About Life Experiences scale (Bluck & Alea, 2002; e.g., “I think back over my life when I want to learn from my past mistakes”; α = .77). Emotion regulation was measured using the subscales for perception (9 items, e.g., “I am often uncertain about what I am feeling”) and regulation (6 items, e.g., “When I am afraid of something, there is little I can do about it”) of one’s own emotions from the German-language Emotional Competence Questionnaire (Freudenthaler & Neubauer, 2005; α = .83). Empathy was assessed by the Empathic Concern subscale of the Interpersonal Reactivity Index (Davis, 1983; α = .68).

Our *open-ended measure of the MORE resources* was an interview about a difficult interpersonal conflict from the participants’ past. As explained earlier, we propose that the MORE resources influence how people reflect upon past life challenges. Therefore, we assumed that participants’ levels of the MORE resources should manifest themselves in the way they talked about such experiences. Their accounts of a conflict should enable us to evaluate their willingness and ability to question their own position and take the opponent’s perspective in retrospect. In the interview, they were first asked to make a list of serious interpersonal conflicts they had encountered. Then, they selected the most difficult conflict they wanted to talk about. They were asked to freely narrate the event and then answer questions concerning what had happened later, how they and their opponent had felt at the time, how they felt about the experience now, and whether they had learned something from it. The interview transcripts were rated by two trained student raters for each MORE resource using 4-point scales from 0 = “no indication of the resource” to 3 = “extraordinary level of the resource”. Table 1 describes the rating criteria for each MORE resource and illustrates them with quotations from the interviews. Participants were also interviewed about another difficult event from their past, but those interviews were difficult to rate in a reliable way. Therefore, we focus on the conflict interviews in the following.

**Table 1:** Rating Criteria and Sample Quotations for the MORE Resources

Resource	Sample Quotations
*Sense of Mastery:*	
(1) Active engagement: taking control of a situation, changing what can be changed, and acting in accordance with one’s convictions.	“There are things in life that cannot be changed, and then you have to accept them. Sometimes you have the choice, and sometimes you just don’t.”
(2) Acceptance of uncontrollability: awareness and acceptance of the fact that many things in life cannot be changed, being able to let such things happen and to come to terms with them.	“I cannot make right what happened then, but I can do it right this time.”
*Openness:*	
(1) Openness and flexibility concerning new experiences and possibilities.	“In no way do I dare to judge how other people would deal with this, with having a child with special needs.”
(2) Openness concerning people, i.e. tolerance and acceptance of different goals and values. As tolerance is highly socially desirable, a high level of openness is coded only if the participant makes no contrary statements in the interview.	“Seeing my son grow up taught me a great number of things, [including] how accepting one is able to be – seeing that a child is not one’s property but an independent human being, and accepting that his generation is just different from mine.”
*Reflectivity:*	
(1) Complexity: taking contextual, developmental trajectories, and multi-causality into account and trying to see the “big picture” as well as the details.	“And I’ve found that fear is permanently present in our society. All unconsciously, fear is being used to manipulate people everywhere. The church, the medical system, they are all relying on people’s fear, people’s bad conscience…”
(2) Willingness to question one’s own views and behavior and to see one’s own role in difficulties without aiming at self-protection or self-enhancement.	“Now I think that those feelings that my father didn’t appreciate me were just my perception at the time. He probably did appreciate me, but I didn’t appreciate myself.”
*Emotion regulation:*	
(1) Comprehensive perception and description of one’s own feelings, including those that are ambivalent or contradictory.	“Well, talking to others is certainly helpful, but you should not use that to get rid of your feelings. You have to see them through, live through them – even if it’s painful, because it will be better later. You can deal with the issue in a better way later and look at it from a meta-level, so to speak, if you’ve really been through the feeling.”
(2) Being able to manage one’s own emotions as is appropriate and relevant to the situation.	“When I get angry about those little things, I tell myself, no, I will not let this make me angry. It is just not worth it.”
*Empathy:*	
(1) Being able to take others’ perspective and perceive their feelings accurately, and to know how to deal with them, that is, to “regulate” others’ emotions well.	“I guess he probably felt that he was losing his daughter. I think he couldn’t really handle the idea that I am a different person than he thought I was. Probably he was also feeling I rejected him somehow. I can imagine that.”
(2) Prosocial motivation: Willingness to support others out of a caring concern for them.	“Sometimes I just feel a deep compassion for that whole complex system of judging and dismissing one another that goes on between people, and how they cannot get themselves out of that”

*Note:* Resources were rated on scales from 0 (“no indication of the resource”) to 3 (“high level of the resource”). Each resource included two aspects, and level 3 was only coded if both aspects were present in the narrative.

Table 2 shows inter-rater reliabilities for the BWP criteria and the MORE resources. Inter-rater reliabilities for the BWP were comparable to other studies (Glück & Baltes, 2006; Mickler & Staudinger, 2008); the total BWP score had a Cronbach’s *α* of .85. Reliabilities for the conflict interview were in the same range, with a Cronbach’s *α* of .88.

**Table 2:** Inter-Rater Reliabilities for the MORE Resource and BWP Criterion Ratings

MORE Resources	Cronbach’s *α*	ICC
Sense of Mastery	.86	.76
Openness	.70	.51
Reflectivity	.90	.77
Emotion Regulation	.75	.59
Empathy	.73	.53

BWP Criteria		
Factual knowledge	.80	.67
Procedural knowledge	.84	.72
Value relativism	.75	.57
Life-span contextualism	.64	.46
Uncertainty	.68	.52

### Results

We used structural equation models to test our hypothesis that the MORE resources would be significantly correlated to wisdom. Separate analyses were performed for the self-report and the open-ended measures. In both cases, we first fit separate measurement models for the resources and the wisdom measures and then combined the optimized measurement models to test the full structural model.

*Self-report measures.* Table 3 displays the correlations between the self-report measures of the MORE resources and wisdom. Interestingly, there was some differentiation among the MORE resources: there were substantial correlations between mastery and emotion regulation and between empathy and reflectivity, but not across these two groups except for openness, which had significant correlations with both emotion regulation and empathy. Consistent with that, a one-factor measurement model for the self-report MORE measures did not fit the data. A two-factor model with mastery and emotion regulation on one factor, reflectivity and empathy on the other, and openness loading on both factors fit the data well. The correlations among the three wisdom scales were all significant (Glück et al., 2013), and they loaded on one factor.

**Table 3:** Correlations between Self-report Measures of the MORE Resources and Wisdom (N = 150)

	Openness	Reflectivity	Emotion Regulation	Empathy	3D-WS	SAWS	ASTI
Mastery	.052	.115	.459	.041	.197	.367	.483
	(p = .527)	(p = .161)	(p < .001)	(p = .615)	(p = .016)	(p < .001)	(p < .001)
Openness		.117	.358	.364	.573	.429	.427
		(p = .153)	(p < .001)	(p < .001)	(p < .001)	(p < .001)	(p < .001)
Reflectivity			−.024	.258	−.057	.376	.264
			(p = .771)	(p = .001)	(p = .489)	(p < .001)	(p = .001)
Emotion Regulation				−.020	.617	.304	.473^)^
				(p = .812)	(p < .001)	(p < .001)	(p < .001)
Empathy					.261	.414	.296
					(p = .001)	(p < .001)	(p < .001)
3D-WS						.258	.474
						(p = .001)	(p < .001)
SAWS							.601
							(p < .001)

**Fig. 1:**
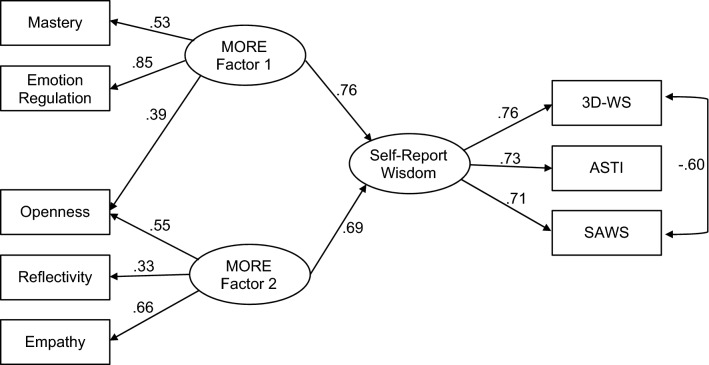
Structural equation models for the self-report measures of the MORE resources and wisdom. Coefficients are standardized estimates

Using these measurement models, we next fitted a structural model predicting the wisdom factor from the two MORE factors. As Table 3 shows, the correlations between the MORE resources and the wisdom scales were all significant except for a zero correlation between reflectivity and the 3D-WS. However, the model did not fit the data well, and the fit remained unsatisfactory after a residual negative correlation between the 3D-WS and SAWS, suggested by modification indices, was permitted, χ^2^ (19) = 80.275, p < .001, GFI = .870, CFI = .839, RMSEA = .164. The remaining modification indices all required correlations between specific MORE resources and wisdom scales, which would have run against the logic of the model. Therefore, the structural model was retained as it was (see Figure 1). In spite of the problems with model fit, both resource factors were strong predictors of wisdom with standardized regression weights of .76 and .69.^1^

**Fig. 2:**
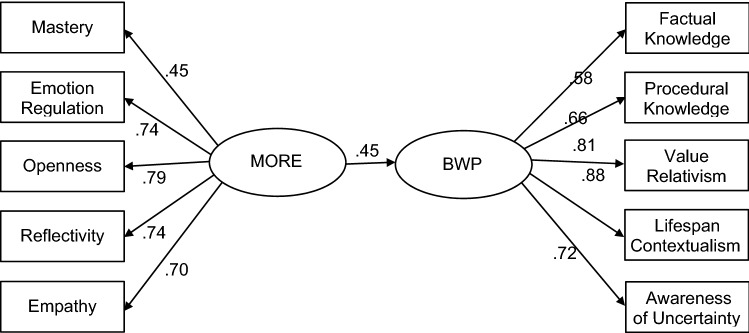
Structural equation models for the open-ended measures of the MORE resources and wisdom. Coefficients are standardized estimates

*Open-ended measures.* Table 4 displays the correlations between the MORE resource ratings of the conflict interviews and the BWP criteria. Here, the measurement model for the MORE resources did not require differentiation into two factors: the one-factor model fit the data very well, and the same was true for the five Berlin criteria. The structural model showed highly satisfactory fit, χ^2^ (34) = 38.324, p = .280, GFI = .915, CFI = .986, RMSEA = .040. It is displayed in Figure 2. As the figure shows, all paths were significant and substantial. The MORE resource ratings from the conflict interview predicted performance criteria in the Berlin wisdom paradigm with a significant regression weight of .45.

**Table 4:** Correlations between Interview Ratings of the MORE Resources and Criteria of the Berlin Wisdom Paradigm (N = 82)

	Openness	Reflectivity	Emotion Regulation	Empathy	Uncertainty	Value Relativism	Lifespan Contextualism	Factual Knowledge	Procedural Knowledge
Mastery	.361	.264	.401	.366	.081	.186	.048	.165	.201
	(p = .001)	(p = .018)	(p < .001)	(p = .001	(p = .476)	(p = .099)	(p = .674)	(p = .145)	(p = .074)
Openness		.635	.473	.499	.179	.254	.256	.301	.301
		(p < .001)	(p < .001)	(p < .001)	(p = .112)	(p = .023)	(p = .022)	(p = .007)	(p = .007)
Reflectivity			.572	.519	.221	.323	.288	.389	.347
			(p < .001)	(p < .001)	(p = .048)	(p = .003)	(p = .010)	(p < .001)	(p = .002)
Emotion Regulation				.592	.182	.290	.191	.247	.317
				(p < .001)	(p = .107)	(p = .009)	(p = .090)	(p = .027)	(p = .004)
Empathy					.179	.225	.137	.189	.265
					(p = .113)	(p = .045)	(p = .226)	(p = .093)	(p = .017)
Uncertainty						.437	.537	.480	.379
						(p < .001)	(p < .001)	(p < .001)	(p = .001)
Value Relativism							.590	.520	.525
							(p < .001)	(p < .001)	(p < .001)
Lifespan Contextualism								.734	.470
								(p < .001)	(p < .001)
Factual Knowledge									.684
									(p < .001)

### Discussion

This study provided a first, cross-sectional test of the hypothesis that the MORE resources are related to wisdom across different measurement methods. The models for both self-report and open-ended narrative measures showed significant and substantial positive relationships between the MORE resources and wisdom. This is not particularly surprising with respect to the self-report data: in our experience, self-report measures of positively valued constructs have a general tendency to covary, if only because they tap into people’s general way of thinking about themselves (Glück, 2018; Glück et al., 2013). Therefore, we were a bit surprised to find the clear dissociation between emotion regulation and mastery on the one hand and empathy and reflectivity on the other. There are at least two explanations for this finding. First, one could argue that emotion regulation and a sense of mastery are about a person’s “internal” way of dealing with experiences, by controlling emotions and by deciding whether to take action or to accept a given situation. Empathy and reflectivity are about taking “external” perspectives: imagining how another person is feeling and thinking about oneself from a self-distanced perspective. On the other hand, the two types of resources may have different developmental timelines: empathy and reflectivity may be predecessors of wisdom, whereas emotion regulation and mastery are acquired with experience and may co-develop with wisdom. We will come back to this point later.

Interestingly, no such dissociation was found for the open-ended data: in the autobiographical interviews about a difficult conflict, participants’ levels of all MORE resources were loading on one factor. Thus, the resources that people utilize when they are reflecting on a concrete event from their past are more closely related than people’s self-evaluations of those resources as traits. Perhaps this is the case because difficult conflicts require both perspective-taking and internal regulation capacities.

In sum, we consider our findings as an encouraging first step in our growing understanding of how wisdom develops. The next step will be to investigate how the resources longitudinally interact with life experiences: the MORE Life Experience Model predicts that the resources that individuals bring to a life challenge influence how they deal with the challenge, but also whether they then grow wiser in its aftermath.

## Lessons We Have Learned

We have gained a number of insights from this study and some smaller “side studies” that we did in connection with it. We will detail these lessons in the following section. First, we discuss lessons that led us to refine the model, then, lessons that extend the model in new directions.

### Optimizing the MORE Life Experience Model

**Single events vs. life phases.** Sometimes, participants had difficulty pinpointing one specific event that led to an important insight or a change in worldview. They might have been struggling with a problem for a long time and then at some point, a relatively minor experience, such as a conversation they had or a book that they read, gave them a whole new perspective on the issue. In addition, even when people felt that a single difficult event had led to a major insight, it was often only in the aftermath of the event, when they had the time and nerves to reflect upon what had happened, that they realized how much they had changed. Thus, we would like to shift the focus away from life-changing events to life-changing *insights* – which often but not always happen in the course of life-changing events. In future studies, we plan to ask participants more directly about the experiences that had the strongest effect on their views about life (see, e.g., Weststrate, Ferrari, Fournier, & McLean, 2018) instead of asking them about difficult events and lessons they derived from them.

**Renaming some resources.** First, while the acronym “MORE” was well suited to convey the general idea that wisdom should be related to “more life experience” as well as to M, O, R, and E/E plus life experience, we may not have chosen the optimal labels for all resources. We would like to change the labels of the “M” and “E” resources as follows.

*Managing uncertainty and uncontrollability.* The original label for the “M” resource, “sense of mastery,” is too closely related to concepts like self-efficacy and internal control beliefs. It captures the trust that wise individuals have in their own ability to master whatever may happen in their life, but it may not convey their above-average awareness of the unpredictability and uncontrollability of human life. We consider both sides to be equally important: wise individuals neither overestimate nor underestimate their control and knowledge about the world. As mentioned earlier, the psychological literature suggests that most people overestimate the control they have over their life and that these control illusions are actually beneficial to their well-being (Peterson & Bossio, 2001; Taylor & Brown, 1988). On the other hand, underestimating one’s control has been related to learned helplessness and associated with depression (Seligman, 1975); thus, there may also be people for whom wisdom comes from realizing that they actually do have control over important parts of their lives. However, as the majority of people tends to overestimate their control and other conceptions of wisdom have also included awareness of uncertainty as a criterion (Baltes & Staudinger, 2000; Grossmann, 2017), we prefer to emphasize in the new “M” that wise individuals are fully aware of the limitations of their control and knowledge, but able to manage this awareness well.

*Emotional sensitivity and regulation.* The original labels for the “E”, emotion regulation and empathy, were somewhat imprecise. Emotion regulation is often used for all phases of perceiving and managing one’s own feelings (e.g., Gross & Thompson, 2007). Empathy is usually distinguished from sympathy in that it refers to being aware of others’ emotions without necessarily sharing them. Our conception of the “E” resource is meant to convey that wisdom involves both an attentiveness and sensitivity to the feelings of oneself and others and the ability to regulate them, so as to remain (relatively) calm and to calm down others in challenging situations. Thus, the distinction between the two aspects of the “E” resource is no longer between the self and others but between (a) sensitivity to emotions, which involves being attentive to one’s own and others’ feelings and taking them seriously even if they are unwanted, and (b) regulation of one’s own and others’ emotions as a context requires, which includes the ability to maintain one’s calm even in emotionally challenging situations. As discussed later, sensitivity may actually be an early predecessor of wisdom like openness or empathy, whereas emotion regulation is more of an acquired competence that is learned with experience over the lifespan.

**Differentiating reflectivity.** One important step forward in our work has been specifying more precisely what kind of reflection about experiences can foster wisdom. Virtually every theory of wisdom involves some aspect of reflection or reflectivity, and virtually all authors agree that it is not having had certain experiences per se, but having reflected upon them that leads to wisdom (e.g., Ardelt, 2005; Glück & Bluck, 2013; Staudinger, 2001; Webster, 2007). However, what authors mean by reflection varies considerably. For example, Webster (2007) defined his subcomponent of reminiscence and reflectiveness as “seeking to understand and derive insight from both our mistakes and successes“ (p. 168) whereas Ardelt (2003) defined her reflective dimension as “looking at phenomena and events from many different perspectives to develop self-awareness and self-insight, [a practice that] will gradually reduce one’s self-centeredness, subjectivity, and projections, and increase one’s insight into the true nature of things, including the motivations of one’s own and other people’s behavior” (p. 278). These two conceptions touch upon somewhat different aspects of reflection; in fact, we found a low but significant *negative* correlation between them (Glück et al., 2013).

How do we understand reflectivity (originally called “reflective attitude”) in the MORE Life Experience Model? In a way, our conception combines Ardelt’s and Webster’s ideas: we believe that thinking back upon experiences is necessary but not sufficient for developing wisdom; *how* one thinks about them matters as well. In line with Ardelt’s characterization, wise individuals reflect upon experiences with the aim of gaining insights and learning more about themselves and life in general. Weststrate and Glück (2017) distinguished two forms of reflection in the interview transcripts from the study described earlier. *Exploratory processing* is an analytical and interpretive way of reflecting about life events that emphasizes meaning-making (i.e., extracting lessons and insights), complexity, and growth from the past. For example, a participant in our study who had a son born with severe mental disabilities said, “I learned trust and acceptance. I am still learning. I am learning the whole time. I very often say that my oldest son is my greatest teacher. […] I just realize that accompanying my son’s life, I am in a constant learning process… I think this has given me strength.” *Redemptive processing*, on the other hand, describes the tendency to transform an initially negative experience into an emotionally positive one, leading to sense of emotional closure and event resolution. A participant who had survived cancer said, “I have a very positive attitude. I thank my organs every day for working well. […] in retrospect, I am glad that I had cancer. […] Feelings of gratitude… I do not think about the cancer itself anymore. That is done. It is in the past. It doesn’t make sense to give in to the fear that it could come back.” (Weststrate & Glück, 2017, p. 807)

Studies have shown that exploratory processing of negative experiences is related to psychological maturity, while redemptive processing is related to happiness and well-being (e.g., King, Scollon, Ramsey, & Williams, 2000; Lilgendahl & McAdams, 2011; Pals, 2006). In our data, exploratory processing was correlated with wisdom, whereas redemptive processing was associated with well-being (Weststrate & Glück, 2017). Thus, wisdom-fostering reflectivity is exploratory in focus, aimed at learning about life in its complexity, and not redemptive, aimed at achieving closure and feeling better.

**Manifestational vs. developmental resources and their different developmental timelines.** In our original model, we assumed that the same resources foster the manifestation of wisdom during life challenges and the development of wisdom from life experiences: a person higher in the MORE resources would deal with a difficult situation in a wiser way and would also be more likely to grow even wiser from that experience. We still believe in the first assumption: a person dealing wisely with a difficult situation will be able to manage uncertainty and uncontrollability, open to alternative views, reflective of his or her views and behaviors, and sensitive to his or her own emotions and those of others involved and able to regulate them as the situation requires. The idea that these same resources also foster the development of wisdom, however, needs some differentiation. Both theoretical considerations and the dissociation we found for the self-report measures suggest that the resources have different developmental trajectories and different ways of interacting with wisdom in the course of development. Some of the resources – especially openness and emotional sensitivity – may be relatively early predecessors of wisdom, already present to individually different degrees in children. Reflectivity is probably learned from experience, starting early on – parents and caregivers can be models of critical self-reflection (or of defensiveness and denial). On the other hand, learning to manage uncertainty and uncontrollability probably requires relevant life experiences. It is therefore most likely to develop as people move from the growth-oriented, self-confident, expansive mindset of adolescence and young adulthood towards a more balanced view of their own power and its limitations in middle adulthood, by which point most individuals have experienced many life challenges. Similarly, while most people learn the “basics” of emotion regulation in childhood, extraordinary levels of this resource may develop in the course of adulthood as people are faced with more difficult emotional challenges such as divorce or serious illness. There are certainly individual differences in the developmental trajectories of all the resources depending on people’s individual experiences. But generally, people at different ages are likely to show somewhat different constellations of the MORE resources.

Importantly, we also believe that the resources interact dynamically with one another. Ideally, they foster each other’s development over time (Glück & Bluck, 2013). For example, if a child who is highly empathetic and emotionally perceptive also has the cognitive resources and environmental modelling and support necessary to acquire reflectivity, he or she is likely to develop effective emotion-regulation skills and an awareness of the limitations of his or her control. These skills, in turn, may enable him or her to become an extraordinary source of support and advice for people in need without burning out emotionally in the process.

**Optimal vs. maximal levels of the resources.** We have also found that for all five MORE resources, the optimal level may not be the maximum possible. It is possible to be so aware of uncertainty and uncontrollability that one becomes helpless, so open to others’ views that one cannot hold one’s own positions, so self-critical that one loses any self-confidence, so sensitive to others’ feelings and concerned about acquiescing them that one sacrifices one’s own well-being. Thus, while for most people becoming wiser means gaining a bit more distance from themselves and learning to take others’ perspectives, for others it may mean building trust in their own feelings, standing by their own values, and taking care of their own needs as well as those of others. Wisdom is a matter of balance more than extremes, and it manifests itself in the way individuals deal with specific, contextualized problems where optimal solutions may not always be possible (Sternberg, 1998).

### Extending the MORE Life Experience Model

**Other potential resources.** One question that we have repeatedly discussed is whether the original five MORE resources are really the most important possible ones. Even if, as we showed earlier, the relationships between them differ somewhat by method of assessment, they are related both empirically and conceptually. In fact, we believe that in their dynamic developmental interaction they form a kind of “self-reinforcing syndrome”. For example, openness and empathy are likely to reinforce a self-reflective attitude, and such an attitude is likely to foster the development of emotion regulation skills, which may again help people remain open to others’ perspectives. It is an interesting question whether this “wisdom syndrome,” which we imagine as a kind of general mindset, includes other attitudes and capacities as well, some of which may even be more specific to wisdom than the ones we have described.

In her master’s thesis, our project member Lara Dorner drew upon the literature on growth in psychological and psychotherapeutic contexts to identify other growth-fostering resources that would also seem relevant to wisdom (Christopher, 2004; Curnow, 1999; Deci & Ryan, 2008; Joseph & Linley, 2005; Jung, 1971; Kramer, 1990; Labouvie-Vief, 1990; Levenson, 2009; Linley, 2003; Pascual-Leone, 1990; Rathunde, 2010; Rogers, 1964; Tedeschi & Calhoun, 2004). She identified six resources that came up across various conceptions of growth (Dorner, 2012). The arguably most important ones are process orientation and self-integration. *Process orientation* is a view of life as continuous learning and growth. Process-oriented individuals know that change is inherent in life and that negative experiences are unavoidable; rather than avoiding these challenges and contradictions, they are open to them and embrace the insights they bring with them *Self-integration* is perceptiveness to and acceptance of one’s own emotions, intuitions, and physical sensations. Self-integrative individuals do not suppress or ignore these perceptions, but are attentive to and accepting of them, even if they run against their ideal of how they would like to be. They aim at integrating even complex and contradictory facets into their own self-concept, which leads to a continually more complex view of the self. *Acceptance and trust* is a general attitude toward life that is able to look at things and let them happen, trusting that things will be okay, or if they are not, one will be able to deal with them, instead of constantly needing to take action and control. *Self-determination* is a way of living one’s life that takes one’s own individual needs and personality into account, follows one’s intrinsic motivations, and does not care about external evaluations or reinforcements. Self-determined individuals take responsibility for their actions as they act in accordance with their own self, and value the autonomy and authenticity of others as well. *Self-transcendence* is a way of experiencing the world that is not centered on one’s own self. Self-transcendent individuals do not feel the need to evaluate others, do not feel threatened when others disagree with them or prove them wrong, and do not depend on the admiration of others. They are compassionate and unselfish as they feel deeply connected to others and the world at large.

These six resources have been shown to be characteristic of growth processes that happen as individuals grow from difficult experiences both inside and outside psychotherapeutic contexts. They have some obvious conceptual relations to the MORE resources (for example, self-integration is related to emotional sensitivity, and process orientation to managing uncertainty), and we find indications of them in our interview transcripts. Thus, they are likely to be facets of the “wisdom-resource syndrome” as well. We selected the original five MORE resources because they were relatively close to established psychological concepts, which made it easier to conceptualize and measure them. It is an open question for future conceptual and empirical work whether additional resources might be added to the model.

**Situational variability of wisdom.** One of our most important insights concerns the question whether wisdom is a stable personal trait or a more fluctuating phenomenon. The idea that people’s wisdom varies across situational contexts has been supported by experimental research (overview in Grossmann, 2017) as well as by studies showing that most people can recall situations in which they did something wise (Bluck & Glück, 2004; Glück et al., 2005). Our research suggests, however, that there is variation not just in how wisely people act across different situations, but also in how wisely they reflect upon past experiences. As described earlier, participants in our study were interviewed about two different experiences: a conflict and a difficult event. We did not only have raters who evaluated the interview transcripts for the MORE resources, but also raters for wisdom, as we thought that it might be possible to measure wisdom by interviewing participants about life challenges. Trained student raters evaluated each transcript concerning the components of three different wisdom models: the Three-Dimensional Wisdom Model (Ardelt, 2003), the Berlin wisdom paradigm (Baltes & Staudinger, 2000), and the Bremen wisdom paradigm (Mickler & Staudinger, 2008). A fourth group of so-called “lay raters” rated the transcripts for wisdom using their own understanding of wisdom. Interestingly, the correlations within each interview suggested that the different wisdom conceptions tap rather similar characteristics: the average correlation was .73 within the conflict interview and .69 within the difficult-event interview. However, the correlations across the interviews suggest much less commonality with an average of .31 (Glück, 2018). Thus, a participant might well have talked very wisely about a conflict from her past but much less wisely about the other difficult event and vice versa.

Much recent research has shown that wisdom varies by situations – the same person may act very wisely in one situation and much less wisely in another. In other words, wisdom is not only determined by a person’s stable personality, but also by situational context (Grossmann, 2017). Our findings suggest that wisdom varies even when the external context of life reflection is held constant: people who are talking about two different situations in the same interview room, with the same interviewer, may still be far wiser about one situation than about the other. Thus, how wisely we are able to think about a past experience varies as well. Different experiences have different meanings for us, they happened in different life phases and taught us different lessons. How wisely we think about them may also depend on how much we have thought about the event before, who we talked to about it, what kind of responses got from others as we talked about it. The stories we make of our past experiences are often constructed in close contact with others; thus, others may have a strong influence on how much wisdom we can gain from an experience. This insight, together with some others, has led us to think more generally about the role of interpersonal resources for wisdom.

**The important role of interpersonal resources.** We began to notice the importance of external resources early on in our study. In particular, Susanne König, a doctoral student and interviewer in the research project, noticed that wisdom nominees seemed to be talking about gratitude far more often than other participants did. Eventually she wrote her dissertation on the relationship between wisdom and gratitude, demonstrating that, indeed, wisdom nominees far more often mentioned spontaneously that they were grateful for something or someone (König & Glück, 2014). Asking the participants what they were most grateful for, she found four categories that were mentioned more often by wisdom nominees: life in general with all its ups and downs, their health, their faith, and their partners. Given that most of them were middle-aged and older adults who had been in their relationships for a long time, one would not necessarily expect them to still feel gratitude for having their partner. One participant described her relationship as the best “event” in her life: “… he feels it when I’m not feeling good, and I can talk to him about it, and yes, I am very grateful that I have such a wonderful relationship. I am happy and grateful that I have him.”

Beyond intimate relationships, we also saw the importance of other people for wise individuals in an ethnographic study that another project member, Katja Naschenweng, carried out (for other ethnographic work on wisdom see Edmondson, 2005, 2013). She wanted to study the small tribe of wise individuals just as one would an indigenous people in a distant corner of the world: by observing how they live their lives. She did so with five particularly wise participants of our study. Among several interesting commonalities she found between those quite different people, one was that while they lived somewhat contemplative lives in quiet places, they had not at all turned away from the outside world: they used media actively and selectively, they were very interested in art, literature, and philosophy, and they valued their active social lives. They considered their partners, family, and friends as important sources of not just happiness, but also insight. One participant said, “You need people with whom you can discuss issues, not just the usual blah-blah. We talk about things that are really important to us. I grow through my friendships and relationships. Sometimes I really want to be challenged in those conversations” (see also Weststrate & Glück, 2017).

In sum, these findings drew our attention to the importance of external, especially interpersonal resources for wisdom. How much and with whom we talk about our experiences and what we make of them may be as important as our internal ways of reflecting upon them. There may be different ways of telling stories that parallel the different forms of reflection that we have identified. Sometimes we talk about an experience for redemption: we know exactly what we want the listener to say to make us feel better, and we choose our audience and tell the story so as to elicit that reaction. At other times, we want to explore, to get to see a viewpoint, to perhaps gain a new understanding. This form of storytelling is clearly more likely to lead to new insights. Perhaps wise people are less reluctant to explore their own experiences by talking about them in this exploratory way than most of us are. This idea may suggest a Vygotskyan perspective (e.g., Kozulin, 2014) on the development of wisdom.^2^ Perhaps there is a “zone of proximal development” for wisdom in the sense that people’s previous life experiences and internal resources determine the extent to which they can grow towards wisdom if they get the right external feedback. A mentor, psychotherapist, or simply a wise friend may be able to open up new perspectives on an experience or situation that may not only help them to resolve a problem but also to grow wiser (Igarashi, Levenson, & Aldwin, 2018).

### Conclusion: The MORE Life Experience Model 2.0

What have we learned so far? A lot, we believe. First of all, our general idea that wisdom develops through a dynamic interaction between experiences and resources has remained unchanged. We have refined some aspects, such as the labels of some resources or our understanding of reflectivity, and broadened our perspective in some ways by including longer life phases and external resources. Thinking about the MORE Life Experience Model has also helped us gain a better understanding of what wisdom itself may be. Some researchers have argued that wisdom is essentially a form of complex, deep and broad knowledge (Baltes & Staudinger, 2000; Sternberg, 1998). Others believe that wisdom is not knowledge but a personality type (Ardelt, 2003). Both these conceptualizations are rendered somewhat incomplete by recent findings that wisdom varies by situation (Grossmann, 2017). Thinking about our model, we have come to believe that wisdom is both: deep, personal, experience-based knowledge about life that is acquired through and goes along with a certain mindset: the willingness and ability to take a broad, non-self-centered perspective on life with the goal of understanding it in all its complexity. People who have this mindset are more likely than others to learn more about life and accumulate wisdom-related knowledge over time, and they are more often able to deal with difficult situations wisely. How we can foster this mindset in human beings may be one of the most crucial questions for humanity at this point.

## Footnotes

1 A potential problem with this analysis was the high degree of conceptual overlap between the MORE Life Experience Model and the subscales of the 3D-WS and the SAWS. The 3D-WS includes a compassionate and a reflective dimension, and the SAWS includes subscales labeled openness, emotional regulation, and reminiscence and reflectiveness. Therefore, we also ran a model that only included the ASTI, which conceptualizes wisdom as self-transcendence and assesses aspects that are relatively distant from the MORE resources (see Koller, Levenson, & Glück, 2017). To have more than one indicator, separate ASTI scores were computed for even and uneven item numbers (*r* = . 78, *p* < .001). This model had a far better fit than the first model, χ^2^ (12) = 33.199, p = .001, GFI = .946, CFI = .933, RMSEA = .109. The standardized regression weights were .82 for the “crystallized” and .57 for the “fluid” MORE resources.

2 We are grateful to an anonymous reviewer for pointing this out!
